# Vestibular evoked myogenic potentials using low frequency stimuli

**DOI:** 10.1590/S1808-86942011000600005

**Published:** 2015-10-19

**Authors:** Aline Cabral de Oliveira, José Fernando Colafêmina, Pedro de Lemos Menezes

**Affiliations:** 1 PhD – Medical School of Ribeirão Preto-University of São Paulo (USP). Professor – Federal University of Sergipe; 2 Associate Professor - Medical School of Ribeirão Preto-University of São Paulo (USP); 3 PhD –University of São Paulo (USP). Adjunct Professor – State University of Health Sciences of Alagoas. Federal University of Sergipe

**Keywords:** evoked potentials, evoked potentials, motor, vestibular nerve, vestibule, labyrinth

## Abstract

**Abstract:**

Vestibular evoked myogenic potentials are vestibulocervical reflexes resulting from sacculus stimulation with strong intensity sounds. Normality parameters are necessary for young normal individuals, using low frequency stimuli, which configure the most sensitive region of this sensory organ.

**Aim:**

To establish vestibular evoked myogenic potential standards for low frequency stimulation.

**Material and Method:**

Vestibular evoked myogenic potential was captured from 160 ears, in the ipsilateral sternocleidomastoid muscle, using 200 averaged tone-burst stimuli, at 250 Hz, with an intensity of 95 dB NAn.

**Case Study:**

Clinical observational cross-sectional.

**Results:**

Neither the student's t-test nor the Mann-Whitney test showed a significant difference in latency or vestibular evoked myogenic potential amplitudes, for *p* ≤ 0.05. Irrespective of gender, we found latencies of p13-n23 and p13-n23 interpeaks of 13.84 ms (± 1.41), 23.81 ms (±1.99) and 10.62 ms (± 6.56), respectively. Observed values for amplitude asymmetry between the ears were equal to 13.48% for females and 3.81% for males.

**Conclusion:**

Low frequency stimuli generate vestibular evoked myogenic potentials, with adequate morphology and amplitude, thereby enabling the establishment of standard values for normal individuals at this frequency.

## INTRODUCTION

Vestibular Evoked Myogenic Potentials (VEMP) are cervical-vestibular reflexes, arising from the stimulation of the sacculus with high intensity sounds^1^,^2^. The responses are collected from the neck muscles, by means of a surface electrode^3^ and may be utilized for vestibular function assessment, specifically that of the sacculus, the lower vestibular nerve and/or the vestibular nucleus^4^, ^5^, ^6^.

VEMP may be present in the region of the frequencies between 100 and 3200 Hz^7^. In human beings, the VEMP is sensitive to different stimulation frequencies, with a greater sensitiveness towards low sounds^7^,^8^. Stimuli in frequencies near 500 Hz have higher response amplitudes, when compared to recordings done with stimuli at 800 Hz^7^,^9^. Some authors^10^ noticed a greater response sensitivity of these responses between 250 and 500 Hz, and other authors^7^ found it between 200 and 400 Hz. The click stimuli are still broadly discussed in the literature, which generate reliable responses, despite stimulating regions between 1000 and 4000 Hz^1^.

In humans, the greater sensitivity towards the low frequencies may be due to the amount of mass in the human sacculus, which is greater than in smaller species and, because of that, they have greater saccular sensitivity for higher frequencies, such as a cat, for example^7^,^11^.

Although the low frequencies are the most adequate to capture VEMP, there are still no normality standards for young healthy individuals. Thus, we aim at standardizing the vestibular evoked myogenic potential for low stimulation frequencies.

## MATERIALS AND METHODS

This study was based on Resolution # 196/96 from the National Health Council, and it was approved by the Ethics in Research Committee of the University where the data was collected, on March 04, 2009, # 1010.

The data was collected from 80 normal volunteers (160 ears), 40 women and 40 men, all bellow 35 years of age (age range between 18 and 31 year, with mean age of 21.28 ± 2.90 years) with auditory thresholds equal to or lower than 20 dBHL for the frequencies between 250 and 8000 Hz for the pure tone audiometry. These individuals did not have a medical history of auditory disorders or vestibular problems, and were allocated by spontaneous demand to the Audiology Ward of the institution, where the data was collected. The VEMP tests were carried out with a specific device used to collect this potential, developed in Brazil, in the Acoustic Instrumentation Lab (AIL) of UNCISAL and at the Center of Instrumentation, Dosimetry and Radioprotection of the FFCLRP-USP (CI-DRA), which is made up of biological amplifiers, filters, electrical protection systems and a logical system which enables a deep investigation of the VEMP^12^.

The recording was made by means of disposable surface electrodes of silver and silver chloride (Ag/AgCl) type. The active electrode was placed in the upper half of the sternocleidomastoid muscle, ipsilateral to the stimulation; the reference electrode on the upper border of the ipsilateral sternum, and the ground electrode on the mid line of the forehead.

After placing the electrodes, we assessed the impedance between the non-inverter electrodes and the ground electrode, and between the inverter electrodes and the ground one. This way, we had an impedance between the electrodes of up to 3 kΩ and that of each electrode alone was 5 kΩ.

In order to record VEMPs at the sternocleidomastoid muscle, the patient must remain seating down, with a maximum head rotation to the contralateral side to the stimulus and maintain the tonic contraction of the muscle around 60 to 80 μV, which was controlled by means of surface electromyography. The stimuli, presented by means of ER-3A in-the-ear phones, started by the right afferent and, later on, was repeated by the left afferent. The responses were confirmed, in other words, recorded twice on the right side and twice on the left side.

In the VEMP test, we used 100 tone burst stimuli, in the frequency of 250 Hz (10ms duration- ascent: 4ms, plateau: 2ms, descent: 4ms), 5 Hz rate, intensity of 95 dB HLn, using a band pass filter of 5 to 2.200 Hz. The recordings were carried out in 50ms windows.

The p13 latency was defined as the positive polarity of the biphasic wave which appears at approximately 13ms, and the n23 latency is defined as the negative polarity of the biphasic wave, which appears, in average, at 23ms.

Amplitude was analyzed by means of calculating the index of interaural asymmetry, which shows the subtraction of the amplitude of the responses from the right muscle from those from the left muscle, in module, divided by the summation of the amplitude of these responses, multiplied by 100. The amplitude from each muscle (right or left) must be calculated by the p13-n2^3^,^8^ amplitude interpeak value.

We used the PASW Statistics data editor software, version 17.0, for the analysis of data, and the sample normal values were found with the Shapiro-Wilk test. We employed the Student-t test independent for the samples which had a normal curve and the Mann-Whitney test, for the sample with abnormal curves. The values were considered significant for *p* ≤ 0.05 and the alpha value used was 0.1.

## RESULTS

VEMP was recorded, with proper amplitude and morphology in 100% of the sample studied. The data will be initially presented per ear and per gender.

On Table 1, we can see that for females, there were no significant differences between the right and left sides as to p13 and n23 latency parameters, except when we see interpeak latencies for *p* values below 0.05.

**Table 1:** VEMP latency values for females, per ear.

		Latência (ms)		
		P13	N23	P13-N23
Right Ear	Mean	13.80	23.58	9.78
	Standard deviation	1.02	1.60	1.53
Left Ear	Mean	14.15	23.56	15.07
	Standard deviation	1.46	2.03	10.34
	*p*-Value	0.25^a^	0.92^a^	0.003^a^

a Independent Student-t test.

For men, in none of the VEMP parameters (p13, n23 and p13-n23 latencies) we found a statistically significant difference between the two ears (Table 2).

**Table 2:** VEMP latency values, for males, by ear.

		Latency (ms)		
		P13	N23	P13-N23
Right Ear	Mean	13.63	23.79	10.16
	Standard deviation	1.31	2.02	2.12
Left Ear	Mean	13.76	23.87	10.11
	Standard Deviation	1.53	2.11	2.15
	*p*-value	0.71^a^	0.87^a^	0.78^b^

a Independent Student-t test;

b Mann – Whitney test.

There were higher latency p13, n23 and p13-n23 values for females, except for the latency parameter of n23, which was higher for males. Using the independent t Student test or the Mann-Whitney test, we did not find differences between the genders, considering a *p*-value below 0.05 (Table 3).

**Table 3:** VEMP latency values per gender.

		Latency (ms)		
		P13	N23	P13-N23
Females	Mean	13.98	23.53	11.85
	Standard Deviation	1.27	1.79	6.10
Males	Mean	13.70	23.83	10.13
	Standard Deviation	1.42	2.05	2.12
	*p*-value	0.22^a^	0.55^b^	0.14^b^

a Independent t-Student test;

b Mann – Whitney test.

On Table 4 we can see normality values (means, standard deviations and maximum thresholds) regarding the p13, n23 latencies to the p13-n23 interpeak values for the sample studied, regardless of gender.

**Table 4:** Reference values for the VEMP latency parameter, regardless of gender.

	Latência (ms)		
	P13	N23	P13-N23
Mean	13.84	23.81	10.62
Standard deviation	1.41	1.99	6.56
Limit (+ 2 standard deviation)	16.66	27.79	23.74

a Independent t-Student test; b Mann – Whitney test

We calculated the interaural asymmetry index for the amplitude, and we found values equal to 13.48% and 3.81%, for females and males, respectively.

## DISCUSSION

The sternocleidomastoid was the muscle chosen to record VEMPs from, having seen that its responses are more homogeneous than the ones registered from other muscles, and it is the one most used today^8^,^13^,^14^.

The placement of the electrodes was similar to what was done in most of the studies ^8^,^13^,^15^,^16^ and, during potential registering, the patient should maintain a tonic contraction of the muscle in around 60 to 80 μV, in an attempt to reduce the interferences from the amplitude^3^.

We chose the tone burst stimulus in 250 Hz, given that VEMPs may be registered in the region of frequencies between 100 and 3200 Hz^7^,^13^,^17^, the responses would be more effective in the low frequency regions (≤1,000 Hz)^3^,^8^.

Moreover, although studies have shown that the cells from the sacculus respond better to low frequency stimuli, there are no studies to standardize the VEMP with 250 Hz stimuli, not even in the international literature. The paper published with a proposal to standardize values for the Brazilian population uses 1,000 Hz^18^ tone bursts. Thus, in the present study we found VEMP in all subjects assessed, with well-defined waves and proper amplitudes, corroborating previous studies for stimuli of different frequencies^13^,^17^,^18^.

The intensity of the stimuli was equal to 95 dB HLn, as advocated by most of the studies, which uses intensities equal to or higher than 90 dB HLn^14^,^19^,^20^.

Analyzing the male and female populations, we did not find significant differences for p13 and n23 absolute latencies between the right and left ears, which corroborates previous studies, using stimuli at the frequencies of 1,000 Hz^18^, 500 Hz^21^ and for click stimuli^22^,^23^.

In the sample studied, we did not find differences between the genders, for all VEMP parameters, as per seen in a previous study^24^, for tone burst stimuli of 250, 500, 750 and 1000 Hz. In other studies (500 Hz tone bursts), there were no reports of changes in VEMP for the absolute latency; nonetheless, we noticed differences in the p13-n2^3^,^22^,^25^ amplitudes. In a prior investigation^26^, it was noticed that there are lower p13 latencies, of 0.73ms in average, among women. Nonetheless, there were no differences as far as amplitude was concerned.

Thus, in the present study, we propose standard values for VEMP results, which are in sync with values reported in the literature for other stimulation frequencies. In our data, we noticed a lower p13 value, the most used parameter in the VEMP analysis^24^,^27^.

For the amplitude parameter analysis, we chose to calculate the asymmetry index, because the absolute amplitude values have very little intra and intersubject reproducibility and are dependent on factors such as age, intensity and frequency of the stimulus and muscle tonus contraction. Thus, in the present study, the asymmetry indices found, both for females and males, were lower than 34%,which is in sync with the lack of interaural assymetries^28^.

## CONCLUSIONS

Low frequency stimuli generate vestibular evoked myogenic potentials with proper amplitude and morphology, thus determining normality values for this frequency in young normal individuals.
